# One Health, One Disease

**Published:** 1877-07

**Authors:** P. H. Hayes


### ONE HEALTH, ONE DISEASE.

P. H. HAYES, M. D.

We ourselves sprang in the primitive ages from barbarians who lived not in houses, but they dwelt every man with his wife, his children of all ages, his goats, pigs and calves, under one single shelter, in one single room. This was the earliest and rudest idea of a house and home. Now, can you imagine a man among such a tribe of barbarians, in whose breast a sentiment of affection and purity begins to dawn, and for the sake of these he begins to think it best for his family that the cattle should have a shelter by themselves, and that himself and family had better become a little exclusive and live by themselves in another wigwam. Can you not imagine that such a man should be looked upon as a strange innovator, a disturber of the customs of his fathers and his tribe? Can you not imagine that in a generation or two more, when the whole tribe should have come up to this level, another man, of yet more affection and sentiment, should rise from this level and make for himself and his family a house with apartments for cooking and sleeping entirely separate? And can you not comprehend that this man should in turn suffer persecution, and be called a disturber—one who thinks himself a little above his neighbors, an outlaw indeed upon the customs of his fathers and his tribe? Thus has civilization grown out of barbarism through the inspiration of love and sentiment and the instinct of purity, yet at every advance have those who remained behind persecuted those who rose up and girded themselves and went before to satisfy yet higher aspirations for purity and refined enjoyments. And so it comes to pass in these days that we look back to some of the customs of our fathers, or our fathers' fathers as ludicrous in the extreme, others distasteful, and others still, mayhap, revolting.

But there are changes which do not elevate, and there is sometimes progress which is not forward nor upward, and we are sometimes obliged to return to a semi-barbaric age to realize some of the evils which have come with the wonderfully various inventions which belong to our own time and to our own civilization.

Sidi Hamet told Capt. Riley that the Arabs who live in the desert subsist wholly on the milk of their camels; that those who never take anything else have no sickness nor disorders of any kind. Hamet assured Capt. Riley that it was very common to find Arabs on different parts of the desert five *zille*, or nearly two hundred years old, retaining all their faculties.

The children of Israel, generally estimated at not much less than three millions, subsisted for forty years upon one single food—"manna." It was like, I judge from the description, what might be made of flour and oil, with a little sugar. As this grand dietetic administration was direct from God, and was intended to qualify these people for soldiers, in view of the coming conquest of Palestine, is it not strange that it should seem to have made no impression upon a Christian age?

The Prophet Daniel, at the court of Nebuchadnezzar, with his three companions, lived for three years upon "pulse to eat and water to drink," and at the end of the three years, when they appeared before the King with a host of youths who had fed upon the King's meat, it is declared that "among them all was found none like Daniel, Hannaniah, Mishael and Azariah: therefore stood they before the King."

Dr. J. R. Farre, an eminent English physician, says: "I remember being consulted by the commander of a British vessel, who had been a captive in Algiers before the Algerines were chastised by Lord Exmouth, 1816. On being made prisoner he was immediately put to hard labor on the public works from four in the morning until four in the evening. He was then turned into his cell, and there he always found a loaf of black bread, weighing a pound, and a pitcher of water. This bread was made from the black wheat of Africa and locusts. Absolutely nothing else was given him. This man was a prisoner nine months at hard labor." In answer to Dr. Farre's question, "Did you enjoy good health?" he replied, "Perfect health. I had not a day's illness. I was as lean as I could be, but I was perfectly well." Dr. Farre adds that "as soon as this man returned to England and home fare, he was compelled to consult me as his physician."

A patient of mine, a lady of real character and remarkably good sense, residing in one of our beautiful inland cities, told me that she knew a family, neighbors and friends of hers, suddenly reduced from affluence to poverty, upon which the husband and father soon died, and the poverty of the mother and three children soon became extreme. She told me that she had been there at meal time, and they had positively nothing to eat but bread and molasses. This lady told me that others of her friends had often been at the house, and they all told the same story as to the poverty of the family and the absolute diet of bread and water. In telling the story, she said while they lived in affluence and luxury, they always had the doctor. The mother or some of those children were forever sick, but after the change came the children became robust and healthy, and the doctor's carriage was never seen at the door.

A year ago a man came into my office extremely thin and sallow. I found him in consumption in an advanced stage, both lungs being badly diseased. I put this man at once upon an absolute diet of bread and milk five times a day. He rallied immediately, and though in the second stage of phthisis he gained by December twelve pounds in weight.

A physician residing in New York told me that there was in Brooklyn a very celebrated Dog Doctor, who made it his business to doctor the pet dogs for which New York, at least, has long been celebrated. These dogs are fed by their mistresses on all sorts of dainties, and acquire, so to speak, all sorts of human diseases. Now this celebrated dog doctor's secret was found out, and it was wholly and solely a strict return to the natural and simple diet of nature for these animals.

There is but *one health*, and that begins in perfect digestion, and it is next found in blood, rich and pure, which gives its color in the cheek and its animus to brain and muscle; and, lastly, the one health is found in a pure and vigorous nutrition in all the organs. 'Tis health that makes food delicious to the palate, and gives it the magnificent seasoning of hunger. It is health that makes digestion so light and easy that we do not know we have a stomach. It is health makes exercise a pastime and sleep to be elysium. It is health gives delight in mere existence. There is but one disease, and that is mal-digestion, malassimilation, poor blood, impure blood, spoiled nutrition. I am consulted almost daily by people who seem to themselves to have as many diseases as they have organs in their bodies; and this is true, strictly true, for is not every organ in distress, *dis-ease*, for the want of a rich and pure blood? Headache, neuralgia, the pains of rheumatism and gout are but an outcry of the nerves for good arterial blood. No matter what name a disease takes, it is indigestion, or *mal*-digestion, reporting itself in the brain, heart, lungs, muscles, arteries or nervous system. In the logic of disease there is but one way to reason, and that is from the stomach outward and onward. In the grammar of diseases there is but one *noun*, and all the rest are *adjectives*. Thus: for rheumatism we would say rheumatic indigestion; for gout, gouty indigestion; for scrofula, scrofulous indigestion, and for melancholy, melancholic indigestion. A good stomach is a peace-maker; a bad one a disturber and peace-destroyer. The stomach is the foundation. All cure of chronic diseases comes through the stomach. And the question of diet and regimen has so much to do with any power over diseases, and I have come to appreciate this fact so much more highly of late years, that I am determined now and here to give the substance of my present directions to patients as to this grand element of success in the cure of their diseases.

And, first, I must say a word as to the number of meals to be eaten in the day. I said above, as to the case of the man with consumption, that I ordered him to eat five times a day. I now order all my patients who have weak digestion, and all who suffer from low nervous or wasting diseases, to eat five times a day. In all these cases I find my patients incapable of digesting full meals at all. I therefore say eat half meals as often again. I say to them, "I don't care how little you eat, but eat it *regularly* five times a day." I found the habit of these patients was to rise late in the morning, with a miserable taste in the mouth, eat a light breakfast, and then wait until 12½ or 1 o'clock, and then one of two things was sure to be the result, either they would have waited so long as to become so exhausted for the want of food that they had no appetite for it and could not eat, or else the appetite would be so strong that they were sure to eat too much and groan the rest of the day from a half-paralyzed stomach. Now I direct these patients to rise in the morning as early as a day-laborer, to take at least a short airing—a stroll in the garden, perhaps—before breakfast, then sit down and eat their first meal. By ten o'clock they are certain to feel a genuine hunger, and then they take another meal, or half-meal I should say. At 12½ o'clock, or by 1 o'clock, at least, the appetite will again return, and again at 4½ p. m., and the last meal should be taken at 7 o'clock. Remember, these patients are allowed but one dish, so to speak, at a meal. If it is bread and milk, that is all; if it is rare eggs, that is all, with a slice of bread and butter. There are no courses in this system. Bear in mind that these patients are directed to retire just an hour after finishing the last meal. They are then prepared to obey the counterpart to this direction, and that is to get up early in the morning. I have now a considerable number of patients with wasting diseases, upon this system; and they are gaining in flesh, strength and color. Only yesterday one of these said me, "I don't know, doctor, as I should ever have got an appetite if I had not begun to eat five times a day. I used to wait so long for the regular meal that when the time came

I could not eat anything." Another young lady told me that as soon as she got established on the one-food-at-a-time and five-meals-a-day system, all her chest pains left her, and that now they will return in a milder degree if she is by accident deprived of her regular meal.

In several instances mothers have come to me and told me how nervous and restless and irritable their children were. I have invariably found these children were in school, where, between play and study, they would become hungry and exhausted long before the regular meal-times. Moreover, it should be remembered that the digestion of children is far more rapid than that of adults, and to compel them to wait just as long for their meals is unnatural, not to say cruel. I have again and again put these small children on to five or four meals a day with magical relief from restlessness and irritability.

[CONCLUDED NEXT NUMBER.]

